# Standards for the conduct and reporting of health technology assessments: Ghana reference case of HTA and economic evaluations

**DOI:** 10.1017/S026646232500011X

**Published:** 2025-03-03

**Authors:** Richmond Owusu, Brian Adu Asare, Genevieve Cecilia Aryeetey, Ivy Amankwah, Emmanuella Abassah-Konadu, Godwin Gulbi, Saviour Yevutsey, Sergio Torres Rueda, Joseph Kazibwe, Francis Ruiz, Joycelyn Zeez, Ghana Health Technology Assessment Technical Working Group, Justice Nonvignon

**Affiliations:** 1 School of Public Health, University of Ghana, Accra, Ghana; 2 Ghana Ministry of Health Technology Assessment Secretariat, Accra, Ghana; 3 London School of Hygiene and Tropical Medicine, London, UK

**Keywords:** reference case, health technology assessment, economic evaluation, Ghana

## Abstract

**Background:**

The methods of economic evaluation and HTA should be based on best practices and standards, tailored to unique country contexts that can be systematically applied to inform decisions. This paper outlines standards for the conduct of economic evaluations for HTA in Ghana.

**Methods:**

A five-step process was followed to develop the HTA reference case as a methodological and reporting benchmark. These include (a) a review of literature and evidence synthesis, (b) a review of country policies, (c) a review and adaption of international frameworks, (d) expert/stakeholder consultations, and (e) the development of a methodological framework. A series of stakeholder consultations were done to refine, finalize, and validate the outcomes of the processes to generate a finalized reference case.

**Results:**

The Ghana reference case is made up of 14 components comprising: evidence synthesis, evaluation type, perspectives on cost, perspectives of outcomes, choice of comparator, data sources, outcome measures, discount rate, uncertainty, equity considerations, time horizon, heterogeneity, transparency, and budget impact. These provide methodological considerations and reporting requirements for economic evaluations for HTA. It provides a framework to ensure the best research methods are adopted to harmonize the evidence-generation process with the expectations of policy and decision-makers and ensure that policy decisions are based on uniform evidence.

**Conclusion:**

Recommendations set out in this reference case when followed can provide context-specific evidence to support a rigorous and transparent system for evaluating healthcare interventions and technologies. It will support decision-making, ultimately improving the quality and efficiency of healthcare delivery in the country.

## Introduction

Countries around the world are increasingly recognizing the importance of Health Technology Assessment (HTA) in ensuring the efficient allocation of resources and improving patient outcomes (1). Ghana has embarked on an initiative to institutionalize HTA, aiming to enhance its healthcare system’s effectiveness, efficiency, and equity (2). In Ghana, HTA has been applied to generating evidence to support financing decisions for new interventions, reimbursement decisions, pricing, and streamlining health insurance benefit packages.

Significant progress has been made in HTA institutionalization since its inception in Ghana. Studies such as HTA on hypertension (3) and childhood cancers (4) have been conducted, with the outcomes informing the Standard Treatment Guidelines and reimbursements under the National Health Insurance Scheme (NHIS) respectively. Other HTA-related analysis includes a cost analysis of the COVID-19 vaccine deployment program (5), and an assessment of amoxicillin dispersible tablets, among others. In line with the institutionalization process, the 1st edition of the Ghana HTA Strategy for HTA has been developed which serves as an essential tool in strengthening the science and practice of HTA in support of evidence-based decisions for the health sector. In addition, the 1^st^ edition of the Ghana HTA Process Guidelines has been developed leveraging the evidence-informed deliberative process (6) and guided by the context from lessons learnt from the National Medicines Selection Process in Ghana (7).

The HTA process guidelines define the stepwise approach to the conduct of HTA and the update of HTA recommendations. Work is also ongoing to explore legislation to support HTA conduct and uptake. Despite the numerous HTA projects conducted in Ghana, the country lacks a standard guide for the conduct of economic evaluation for HTA that should serve as a reference case. “The reference case gives a formal statement of accepted methods and assumptions underpinning analyses to which submissions should conform”^1^. It provides a guide for all parties involved in conducting economic evaluation and HTA, including policymakers, researchers, and healthcare providers, and allows them to adopt a consistent and evidence-based approach to decision-making (8). Researchers and policymakers have relied on international guidelines such as World Health Organization guidelines for HTA (9), and International Decision Support Initiative (iDSI) reference case for economic evaluations (10) among others.

Several developing countries including Ghana at various stages of HTA implementation lack context-specific standards for economic evaluation in HTA. The iDSI recommends that due to the differences in the country’s health systems, there is a need to contextualize recommendations for the conduct of HTA to reflect country-specific health system characteristics (10;11). A lack of standardized methods specific to health systems can result in misinterpretation of the findings and pose credibility issues (12). Promoting adherence to reference cases ensures that they serve as a useful resource for researchers and policymakers in global health settings (13).

Although acknowledging the need for flexibility, a consistent methodological approach is required for assessments to facilitate comparisons between health technologies and disease areas. The development of a reference case for economic evaluation and HTA in Ghana would provide the much-needed structure to support the HTA process and guide the conduct, primary analysis, and reporting of HTA. It will also support the HTA Secretariat of the Ministry of Health (MOH) in the planning and management of the conduct of HTA, harmonize expectations of policy and decision-makers and all relevant stakeholders in relation to HTA findings, and ensure that policy decisions based on HTA are based on a uniform and transparent process and in accordance with set standards.

## Methods

In developing the reference case for the conduct of HTA in Ghana, a five-step process was followed to ensure that adequate evidence was provided to generate recommendations. The processes include (a) a review of literature and evidence synthesis, (b) a review of local policies and strategies (c) review and adaptation of international frameworks, (d) expert/stakeholder consultations, and (e) development of methodological framework.

### Review of literature and evidence synthesis

A thorough review of the literature was conducted to gather existing evidence on reference case development methodologies, best practices, and relevant frameworks from both international and local contexts. Peer-reviewed journals and grey literature including institutional Web sites were searched for relevant literature. Also, the review assessed various economic evaluation methodologies. This review served as the foundation for developing a tailored approach suitable for the Ghanaian health system context.

### Review of local policies and guidelines

The reference case was inspired by the Ghana National Health Policy (revised edition, 2020) (14) and the National Medicines Policy (NMP), 3rd edition 2017 (15). The National Medicines Policy (NMP), 3rd edition 2017, defined the policy direction for HTA and associated implementation steps. The reference case is also aligned with the objectives of the 1st edition of the Ghana HTA strategy which seeks to strengthen the science and practice of HTA to inform policy decisions and the 1st edition of the Ghana HTA Process Guidelines. These policies reviewed served as a policy foundation upon which the Ghana reference case was developed.

### Review and adaptation of international frameworks

Recognizing the value of international experience and expertise, existing international frameworks and guidelines for reference case development were adapted to suit the Ghanaian context. The Ghana reference case draws on principles from other reference cases including the iDSI reference case, formerly referred to as the Gates reference case (10), Health Intervention and Technology Assessment Program (HITAP) Process Guidelines (16), and WHO framework on Using Health Technology Assessment for Universal Health Coverage (9). These frameworks were carefully reviewed, and tailored to align with the country’s healthcare system, epidemiological profile, resource constraints, and cultural considerations.

### Expert/stakeholder consultations

To ensure the inclusivity and relevance of the reference case development process, a comprehensive stakeholder engagement strategy was implemented. Key stakeholders, including policymakers, healthcare professionals, researchers, patient representatives, and industry experts, were identified and invited to participate in the development process (see Table 1). Stakeholder consultations, workshops, and focus groups were conducted to gather diverse perspectives, insights, and expertise regarding the specific needs and priorities of Ghana’s healthcare system. The HTA technical working group and secretariat triangulated expertise from health economics, clinical medicine, public health, epidemiology, policy analysis, and health financing. They played a vital role in guiding the reference case development process, synthesizing evidence, providing expert opinions, and ensuring the validity and relevance of the developed recommendations.

**Table 1.** Profile of stakeholder engagement participants

Form of engagement	Number of participants	Profile/organization
Workshop	16	Clinicians/pharmacists – (GHS/Teaching hospitals)
		Policy makers – (MoH)
		Academic – Universities
		Payers - (NHIA)
		Regulators - (FDA/GSA)
		Civil society organization
Focus Group Discussion	11	Clinicians/pharmacists – (GHS/Teaching hospitals)
		Policy makers – (MoH)
		Academic – Universities
		Payers - (NHIA)
		Regulators - (FDA/GSA)
		Advocates - Civil society organization
Expert opinion	5	Policy makers – (MoH)
		Academic – Universities
		Clinicians/pharmacists – (GHS/Teaching hospitals)

Abbreviations: GHS, Ghana Health Service; MoH, Ministry of Health; FDA, Food and Drugs Authority; Ghana Standards Authority; NHIA, National Health Insurance Authority.

Experts at the HTA secretariat developed a draft reference case after synthesizing the evidence and reviewing the local and international framework. A first draft was shared with experts in the technical working group and partner organizations for input and refinement. Suggestions were discussed by the secretariat, technical working group, and partner organizations for consensus about best practices. Further revisions and refinements were made and presented again to the various stakeholders prior to the finalization of the reference case.

### Development of methodological framework

Based on the literature review, stakeholder consultations, and expert input, a methodological framework for reference case development specific to Ghana was developed. This framework outlined the principles, methods, and criteria for conducting HTA evaluations, considering factors such as clinical effectiveness, cost-effectiveness, ethical considerations, equity, and patient preferences. The framework incorporated rigorous methodologies, including systematic literature reviews, economic evaluations, and health outcome assessments.

## Results

### Standards for conducting and reporting HTA in Ghana

**Figure 1.**
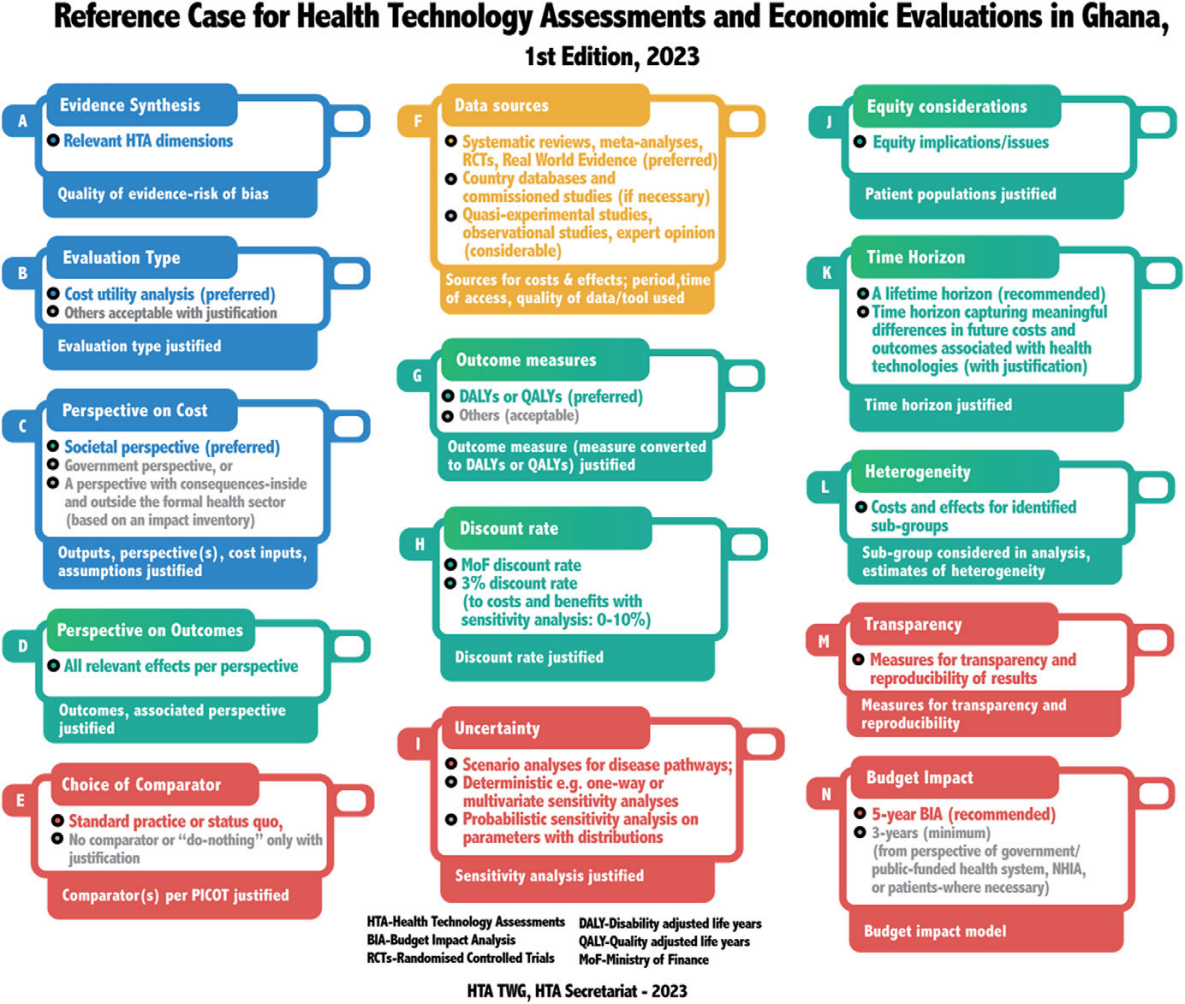
Elements of Ghana Reference Case.

Table 2 provides a summary of the various recommendations for the conduct of economic evaluations for HTA in Ghana. These could be applied to new economic evaluation to inform decisions and an adaptation, transfer, or a systematic mapping of existing economic evaluations or HTA to the Ghanaian context. The framework of the reference case elements is presented in Figure 1.

**Table 2.** Summary of recommendations of the Ghana reference case

	Component	Description of methodological considerations	Reporting requirement
**A**	Evidence synthesis	Evidence should be synthesized on the various relevant dimensions of an HTA based on the scope of the HTA and the decision problem.	The summary of evidence on the various dimensions of the HTA and the quality of evidence should be captured, as well as ROB assessments.
**B**	Evaluation type	The preferred evaluation type is a cost-utility analysis (CUA), with the outcomes expressed in terms of Quality Adjusted Life Years (QALYs) gained or Disability Adjusted Life Years (DALYs) averted. Other economic evaluations are acceptable provided a strong justification is made for their adoption.	In reporting the outputs, the reasons for selecting the evaluation type should be clearly stated and justified. A fully executable economic model should be submitted as part of the reporting.
**C**	Perspective on costs	The preferred perspective is the societal Perspective. However, the perspective could be that of the government (defined to include public-funded health system or the National Health Insurance Authority). Depending on the technology being assessed, a perspective could be analyzed to reflect consequences both inside and outside the formal health sector. An impact inventory detailing such consequences may be necessary.	In reporting the outputs, the perspective(s) chosen, various cost inputs, underlying assumptions, and the reasons for selection should be clearly stated and justified.
**D**	Perspective on outcomes	All relevant effects based on the chosen perspective accruing to individuals, the payer, the health system, the government, or society should be included in the outcome analysis, and this should be in line with the decision problem (as framed by the PICOT statement)	In reporting the outputs, the outcomes selected and the associated perspective as well as the approach to the evaluation of the outcomes should be stated and justified.
**E**	Choice of comparator	The choice of comparator should reflect the decision problem (as framed by the PICO statement) and should include a comparison with standard practice or the status quo. Having no comparator (having a comparator as “doing nothing”) should be justified.	In reporting the outputs, the comparator(s) selected, and the associated justification should be stated and justified.
**F**	Data sources	Systematic reviews, meta-analyses, Randomized Controlled Trials (RCTs) and Real-World Evidence are preferred sources of data. However, the use of data from quasi-experimental studies, observational studies, and expert opinion will be considered where appropriate and based on the decision problem. Data from country databases and commissioned studies would be used if necessary.	In reporting data sources for both costs and effects, the effective period of the data and time of access should be clearly stated. The tool used to assess the quality of data should also be stated. The risk of bias (ROB) of the evidence used should be explicitly assessed.
**G**	Outcome measures	The preferred outcome measure of choice should be DALYs averted or QALYs gained. Other outcome measures are acceptable, provided a strong justification is made for their choice.	In reporting, the choice of outcome measure used should be clearly stated and justified. Alternative measures may be converted into DALYs or QALYs.
**H**	Discount rate	The applicable discount rate from the Ministry of Finance should be used. Where that is not available, the discount rate should be the standard 3% rate commonly used in global CEA studies and applied to both costs and benefits. Sensitivity analysis should be used to explore the impact of discount rates between 0 and 10%.	In reporting, the choice of discount rate for both costs and benefits should be clearly stated and justified.
**I**	Uncertainty	Uncertainty should be evaluated using scenario analyses for different disease progression paths; deterministic (for e.g., one-way, or multivariate sensitivity analyses) or probabilistic sensitivity analysis on parameters that have distributions.	In reporting, the choice of sensitivity analysis employed should be clearly stated and justified.
**J**	Equity considerations	Equity implications and issues should be considered, where necessary, during the economic evaluation.	In reporting, all patient populations considered in the analysis should be stated and justified.
**K**	Time horizon	The time horizon or duration should be adequate to capture any meaningful differences in the future costs and outcomes associated with the technologies. A lifetime horizon is recommended. However, a strong justification should be provided when this is not possible or relevant to use.	In reporting, the time horizon or duration that adequately captures all relevant costs and benefits should be clearly stated and justified.
**L**	Heterogeneity	Costs and effects of the health technology on identified subgroups and populations should be considered.	In reporting, all distinct subgroups considered in the analysis should be documented. Estimates of heterogeneity in metanalyses (I2) should be considered.
**M**	Transparency	All measures to ensure transparency and reproducibility of results should be employed.	In reporting, all measures adopted to ensure transparency and reproducibility should be documented.
**N**	Budget impact	The BIA should be conducted over a minimum period of 5 years from the perspective of the government/public-funded health system or the NHIA and patients where necessary.	In reporting, a fully executable budget impact model should be submitted to enable (confidential) third-party validation of the results.

### Evidence synthesis for health technology assessment

Evidence syntheses summarize the current body of evidence on a specific question or query. The general outcome of an evidence synthesis on any specific issue should be largely reproducible and repeatable.

***Methodological recommendation***: Evidence should be synthesized on the various relevant dimensions of an HTA based on the scope of the HTA and the decision problem.

Evidence synthesis should include framing the relevant questions (population, intervention, comparator(s), outcome(s)); searching for evidence of efficacy; appraisal of a systematic review; summarizing the results of a systematic review; searching for additional evidence; assessing the ‘quality’ of evidence; grading of evidence, with due cognizance to the hierarchy of evidence and risk of bias (ROB). Preferably, the quality of evidence should be determined using the GRADE approach (17;18). The Jadad or Oxford Quality Scoring System (19) may also be used to assess the quality of clinical trials or any other acceptable tools for evaluating the quality of evidence. The evidence synthesized should be of value to the dimension under consideration. The evidence synthesis may cover health outcomes that are broad enough to capture all socially valued aspects of health and are applicable across investment types. Where appropriate, the synthesis of evidence can include statistical ‘pooling’ of results. Bias should be assessed and reported as appropriate.

***Reporting requirement***: In reporting the outputs, the summary of evidence on the various dimensions of the HTA and the quality of evidence should be captured as well as ROB assessments.

### Evaluation type

***Methodological recommendation***: The preferred evaluation type is a cost-utility analysis (CUA), with the outcomes expressed in terms of Quality Adjusted Life Years (QALYs) gained or Disability Adjusted Life Years (DALYs) averted. Other economic evaluations are acceptable provided a strong justification is made for their adoption.

The use of a generic measure of outcome such as the QALYs or DALYs makes it possible to compare outcomes from different technologies across different activities in the healthcare sector. Where patient outcomes in the form of QALYs or DALYs are available, a cost-utility analysis (CUA) will be the preferred choice. Where appropriate a cost-effectiveness analysis where outcomes are measured as life-years gained, natural units/intermediate outcomes, or any other relevant outcome, may be considered.

In certain circumstances, a cost minimization or cost-benefit analysis may be conducted. Where convincing evidence is available to show that important outcomes of health technologies are similar, a cost minimization analysis will be considered. The health sector interfaces with other sectors such as food and agriculture, aquaculture, finance, and economic planning as well as trade and industry. These interactions may necessitate a comparison between health and non-health sector interventions to inform decisions, suggesting a potential consideration for a cost-benefit analysis.

***Reporting requirement***: In reporting the outputs, the reasons for selecting the evaluation type should be clearly stated and justified. A fully executable economic model should be submitted as part of the reporting.

### Perspective on costs

***Methodological recommendation***: The preferred perspective is the societal perspective; however, the perspective could be that of the government (defined to include public-funded health system or the National Health Insurance Authority (NHIA)). Depending on the technology being assessed, a perspective could be analyzed to reflect consequences both inside and outside the formal health sector. An impact inventory detailing such consequences may be necessary.

Most economic evaluations are conducted from a public payer, private payer, individual, or societal perspective. The perspective taken is essential in defining the costs, resources, and consequences that should be examined, applying the economic principle of forgone welfare/opportunity cost (economic cost is emphasized over accounting cost). To ensure comparability of analyses, the perspective must be clearly stated so that the costs, resources, and consequences associated with the perspective adopted can be clearly identified for inclusion in the economic evaluation.

The societal perspective is a broad perspective encapsulating the government/ health system, patients, health care providers, etc. Other costs may also be associated with the implementation of a particular health technology. These may include direct and indirect costs to other public sector agencies, patients, or their caregivers because of technology.

***Reporting requirement***: In reporting the outputs, the perspective(s) chosen, various cost inputs, underlying assumptions, and the reasons for selection should be clearly stated and justified.

### Perspective on outcomes

When conducting economic evaluations, selecting appropriate outcome measures is crucial for accurately assessing the costs and consequences of healthcare interventions. The choice of outcome measures should align with the research question, capture the relevant impacts of the intervention, and provide meaningful information for decision-making.

***Methodological recommendation***: All relevant effects based on the chosen perspective accruing to individuals, the payer, the health system, the government, or society should be included in the outcome analysis, and this should be in line with the decision problem (as framed by the Population, Intervention, Comparator, Outcome and Time (PICOT) statement).

For direct health effects, QALYs gained, DALYs averted, life-years gained, and any other relevant measure of health outcome may be used and justified. For non-health effects, outcomes that fall outside the health budget should be included in the analysis.

***Reporting requirement*:** In reporting the outputs, the outcomes selected and the associated perspective as well as the approach to the evaluation of the outcomes should be stated and justified.

### Choice of comparator

The choice of a comparator is a crucial step in every economic evaluation and must represent the decision problem (framed by PICOT if applicable). This is because the costs and effects associated with a particular comparator will be measured, valued, and included in the analysis. Comparative incremental analysis against current practice can then most accurately reflect the true nature of the decision problem facing decision-makers. A comparator that does not reflect the decision problem and policy context will lead to spurious conclusions.

***Methodological recommendation***: The choice of comparator should reflect the decision problem (as framed by the PICOT statement) and should include a comparison with standard practice or the status quo. Having no comparator (having a comparator as “doing nothing”) should be justified.

The preferred comparator for the reference case or standard economic evaluation will be standard/usual/routine care which represents the technology or technologies most widely used in practice (best practice) for example, in accordance with the Ghana Standard Treatment Guideline (STG).

Although it is best practice to include all relevant comparator technologies in a single evaluation, this may be inefficient and burdensome when there are many available alternatives. It is therefore reasonable to select the best comparator by limiting the choice to usual or standard practice also known as routine care/practice or the technology that would most likely be replaced with the introduction of the new alternative, taking into consideration the decision problem. In the absence of an active comparator or not-well-defined standard care, a comparator of ‘no intervention’ may be used in addition to ‘not standard routine care’ as this will provide useful information on the relative benefits of the technology.

In the event where an intervention that is considered as best practice (as defined by evidence-based clinical practice guidelines) differs from routine practice (e.g., as captured by STG), the choice of the comparator should include both the best practice and routine practice. Where only one of them must be chosen, justification should be provided.

***Reporting requirement***: In reporting the outputs, the comparator(s) selected, and the associated justification should be stated and justified.

### Data sources

***Methodological recommendation***: Systematic reviews, meta-analyses, Randomized Controlled Trials (RCTs) and Real-World Evidence are preferred sources of data. However, the use of data from quasi-experimental studies, observational studies, and expert opinion will be considered where appropriate and based on the decision problem. Data from country databases and commissioned studies would be used if necessary.

Consideration will be given to the hierarchy of evidence in the context of data sources. Systematic reviews, and RCTs will be ranked higher than other studies, however, the use of studies such as cohort studies, observational studies, and expert opinion will be considered where appropriate and based on the decision problem. Where commissioned studies are utilized to generate data, the sources should be cited as part of the report. Also, the use of existing country databases is encouraged. Although data access and data availability constraints are common in the Ghanaian context, key assumptions in the use of proxy data sets and modified data sets should be reported on. Sources from grey literature should be reported.

***Reporting requirement***: In reporting data sources for both costs and effects, the effective period of the data and time of access should be clearly stated. The tool used to assess the quality of data should also be stated.

### Outcome measures

Health outcomes should be the emphasis of all health-economic analyses. Therefore, a health outcome measure must be comprehensive enough to capture the most critical and crucial components of health. It should be able to be used consistently throughout the population to various types of health interventions, technologies, and programs.

***Methodological recommendation***: The preferred outcome measure of choice should be DALYs averted or QALYs gained. Other outcome measures are acceptable, provided a strong justification is made for their choice of outcome.

The QALY is typically used in many high-income countries as health state valuations for computing QALYs are readily available. DALYs seem to be the outcome measure of choice for most economic evaluations in LMICs. Where locally relevant QALYs are unavailable, DALYs may be used. Other alternative outcome measures can be adopted where justification is provided. Note that in a cost-benefit analysis, both outcomes and costs are expressed in monetary units.

Although using a measure that captures both length and health-related quality of life and is generalizable across disease states allows for the consideration of opportunity costs across the entire health sector as well as comparisons between investment types, sometimes a disease-specific intervention may be the appropriate outcome measure depending on the scope of the decision problem and generalizability may be irrelevant. Where appropriate, the use of life-years gained, natural units/ intermediate outcomes, or any other relevant outcome may be employed as the outcome measure.

***Reporting requirement***: In reporting, the choice of outcome measure used should be clearly stated and justified.

### Discount rate

Discounting is a procedure for adjusting future costs and benefits to arrive at their present values. Future predicted costs and health outcomes are usually valued at less than present values, and so best practices in economic evaluations usually recommend discounting, although there remains a debate on whether to debate health benefits at the same rate as costs.

***Methodological Recommendation***: The applicable discount rate from the Ministry of Finance should be used. Where that is not available, the discount rate should be the standard 3 percent rate (20) commonly used in global CEA studies and should be applied for both costs and benefits. Sensitivity analysis should be used to explore the impact of discount rates ranging between 0 and 10 percent (20).

***Reporting requirement***: In reporting, the choice of discount rate for both costs and benefits should be clearly stated and justified.

### Uncertainty (sensitivity analysis)

Uncertainty in health economic evaluations may arise as a result of the structure of a model, for example, how health states are categorized or the representation of care pathways; as a result of bias due to selective use of data sources to inform key parameters, for example, estimates of relative efficacy, selection of cost data; or from parameter precision, that is, the precision of the mean parameter values. To ensure the robustness of the results and conclusions of the economic analysis, uncertainty on the outcome of the economic evaluation should be systematically evaluated.

***Methodological recommendation***: For the reference case analysis, uncertainty should be evaluated using scenario analyses for different disease progression paths; deterministic for example, one-way, multivariate sensitivity analyses, or probabilistic sensitivity analysis on parameters that have distributions.

***Reporting requirement***: In reporting, the choice of sensitivity analysis employed should be clearly stated and justified.

### Equity considerations

Equity in health implies that ideally, everyone should have a fair opportunity to attain their full health potential and that no one should be disadvantaged from achieving this potential (21).

***Methodological recommendation**:* Equity implications and issues should be considered, where necessary, during the economic evaluation.

A starting place for all economic evaluations should be to acknowledge and respect both horizontal and vertical equity. Horizontal equity requires that people with like characteristics (of ethical relevance) be treated the same, whereas vertical equity allows for people with different characteristics (of ethical relevance) to be treated differently. Candidate equity characteristics include age, gender, socioeconomic status, availability of alternative therapies, and prevalence of the condition.

The potential benefits, harm, and costs associated with health technology are often unevenly distributed across the population. This may be due to differences in treatment effects; risks or incidences of conditions; access to health care; or technology uptake in population groups. When the intervention can be provided selectively to certain subgroups, then cost-effectiveness information can be presented for each subgroup. Any stratified analysis of subgroups motivated by vertical equity considerations must be explained and justified. Further, groups that are likely to be disadvantaged by the adoption and implementation of the intervention should also be identified, where possible. This may occur, for example, when a change in clinical practice requires that patients be cared for at home rather than at the hospital, thereby shifting costs and burdens to patients and informal caregivers. Given that many decision-makers are concerned about equity, economic evaluations should be presented in a manner that supports equity concerns being reflected in decision-making.

Although the reference case analysis should weigh all outcomes equally (regardless of the characteristics of people receiving the health benefit), analyses should be presented with full descriptions of the relevant patient populations, to allow for consideration of any subsequent distributional and equity-related policy concerns.

***Reporting requirement***: In reporting, all patient populations considered in the analysis should be stated and justified.

### Time horizon

For economic evaluations, the study period should be clearly described and appropriate to the disease and its treatment or health program. The time horizon should capture all meaningful differences in costs and outcomes between the various interventions. The time frame adopted should be clearly stated and its choice justified, with the same time horizon being applied to both costs and outcomes.

***Methodological recommendation***: The time horizon or duration should be adequate to capture any meaningful differences in the future costs and outcomes associated with the technologies. A lifetime horizon is recommended. However, a strong justification should be provided when this is not possible or relevant to use.

A lifetime horizon is usually considered appropriate for HTA, as most technologies have costs and outcomes that impact a patient’s lifetime. This is particularly relevant for chronic diseases. A shorter time frame may be considered when the costs and outcomes relate to a relatively short period of time, such as in an acute infection, and when mortality is not expected to differ between the competing technologies. A decision to use a shorter time frame should be justified and an estimate provided of any possible bias introduced because of this decision.

***Reporting requirement***: In reporting, the time horizon or duration that adequately captures all relevant costs and benefits should be clearly stated and justified.

### Heterogeneity

Economic evaluations should reflect the entire target population as defined by the decision problem; however, it may be necessary in some cases to assess the cost-effectiveness of the intervention in a subgroup of the population.

***Methodological recommendation**:* Costs and effects of the health technology on identified subgroups and populations should be considered.

In conducting an evaluation, potential sources of heterogeneity that may lead to differences in parameter input values across distinct subgroups should be explored. Heterogeneity may result from differences in the natural history of the disease, effectiveness of the interventions, health state preferences, or costs of the interventions. Heterogeneity may result in different decisions with respect to cost-effectiveness among different subgroups. Care should be taken when representing subgroups to ensure that ethical issues are considered before the analysis is undertaken.

The evidence supporting the biological or clinical plausibility of the subgroup effect should be fully documented, including details of statistical analyses. Since the goal of the health system is to maximize the potential for health gain from its finite resources, a stratified analysis that allows cost-effectiveness to be modeled separately for each subgroup may contribute important information to the final advice.

***Reporting requirement***: In reporting, all distinct subgroups considered in the analysis should be documented.

### Transparency

***Methodological recommendation**:* All measures to ensure transparency and reproducibility of results should be employed.

The economic evaluation conducted should be transparent and reproducible. It should adhere to the Consolidated Health Economic Evaluation Reporting Standards (CHEERS) statement (22) for reporting. To maximize transparency, the assessment should include a conflict-of-interest statement in relation to all those involved in the assessment. In assessing evidence, a reproducible search strategy should be employed, and two or more reviewers should be involved in the selection process using a predefined protocol to maximize objectivity. Data used in the analysis should ideally be publicly available or available upon request, and where possible unit cost should be detailed separately to the total costs. Undiscounted, disaggregated cost and outcome data should be presented in addition to providing the aggregated, discounted summaries.

Data sources should be identified using a comprehensive and transparent approach that can be replicated by others and the choice of data sources and methods for analyzing data inputs must be clearly stated. Details of funding partners of the economic evaluation should be disclosed as well as institutions in support.

***Reporting requirement**:* In reporting, all measures adopted to ensure transparency and reproducibility should be documented.

### Budget impact analysis

A budget impact analysis (BIA) is a financial approach designed to estimate, over a specified time period, the financial consequences of adopting a health intervention or technology.

***Methodological recommendation***: The BIA should be conducted over a minimum period of 5 years from the perspective of the government/public-funded health system or the NHIA and patients where necessary.

A budget impact analysis should be submitted along with the economic evaluation of a technology to best inform the needs of the decision-maker; BIA is often complementary to CEA. The outcome of BIA is the net financial impact, which serves the purpose of determining affordability and informing financial planning for new technologies relative to the status quo. Even though different specifications may be used for a BIA, within the context of this guideline, BIA denotes an analysis of the added financial impact of a new health technology for a finite period. The presentation of BIA should reflect a manner relevant to the decision problem and meet the needs of the decision-maker.

A summary of the conduct of BIA from the perspective of the government of Ghana or the National Health Insurance Authority (NHIA) is detailed below:

***Perspective:*** The BIA should be conducted from the perspective of the government/public-funded health system or the NHIA.

***Technology/intervention*:** The technology should be described in sufficient detail to differentiate it from its comparators and to provide context for the study.

***Choice of comparator(s):*** The preferred comparator for the reference case is ‘routine care’, that is, the technology or technologies most widely used in clinical practice in Ghana in the context of the target population. When both CEA and BIA are conducted, the same comparator(s) should be used in both assessments.

***Time frame/horizon**:* The core analysis should estimate the annual financial impact over a minimum of three years assessment and ideally five-year projections should be provided.

***Target population:*** The target population should be defined based on the approved indication for the technology. The size of the target population should be guided by the national incidence and prevalence of the indication/disease. Stratified analysis of subgroups (that have been ideally identified *a priori*) is appropriate. These should be biologically plausible and justified in terms of clinical and cost-effectiveness evidence, if conducted.

***Costing:*** The costs included should be limited to direct costs associated with the technology that will accrue to the government/public health system and NHIA. The methods used to generate these costs should be clearly described and justified, with all assumptions explicitly tested as part of the sensitivity analysis. As costs are presented in the year they are incurred, no discounting is required.

***Budget impact model:*** The budget impact model should be clearly described, with the assumptions and inputs documented and justified. Two primary scenarios should be modeled: the baseline scenario that reflects the current mix of technologies and forecasts the situation should the new technology not be adopted, and the new technology scenario, where it is adopted. The methods for the quality assurance of the model should be detailed and documentation of the results of model validation provided. Key inputs should be varied as part of the sensitivity analysis. The model should be of the simplest design necessary to address the budget impact question using a readily available software package.

***Uncertainty:*** Scenario analyses for a range of plausible scenarios and sensitivity analysis must be employed to systematically evaluate the level of uncertainty in the budget estimates due to uncertainty associated with the model and the key parameters that inform it. for example, the impact on budget by coverage levels. The range of values provided for each parameter must be clearly stated and justified, and justification provided for the omission of any model input from the sensitivity analysis.

***Reporting**:* For the purposes of financial sustainability, budget impact analysis should be conducted. Input parameters and results should be presented both in their disaggregated and aggregated forms with both incremental and total budget impact reported for each year of the time frame. A minimum five-year budget impact model should be submitted to enable (confidential) third-party validation of the results.

In reporting, a fully executable budget impact model should be submitted to enable (confidential) third-party validation of the results.

## Discussion

This paper summarises the recommendations for conducting and reporting economic evaluations and health technology assessments in Ghana as part of the HTA institutionalization framework. The recommendations therein are to serve as a guide for researchers when undertaking economic evaluations and HTA in Ghana. Further, the reference case is also expected to support decision-makers, members of the appraisal subgroup of the HTA technical working group, and reviewers to critically assess the quality of economic evaluations and HTA in Ghana.

Foremost, the Ghana reference case recommends cost-utility analysis (CUA) as the preferred standard form of economic evaluation. CEA is the most common analytic technique used in economic evaluation studies conducted in the health sector of Ghana (23–29). For CEA, the health outcomes are reported in natural units or in terms of clinically defined outcomes. Consequently, comparison across a wide range of diseases and intervention types becomes difficult and does not support decision-making across interventions (30). To standardize economic evaluation studies in Ghana, the reference case recommends undertaking CUA and reporting results using QALYs gained. This is necessary to reduce the variability between analytic techniques that researchers use in conducting various economic evaluation studies in Ghana. The recently published Ghanaian EQ-5D-5L value set (31) is useful for deriving utility weights to inform CUA. This context-specific QALYs will be useful for CUA and comparability of studies across different interventions.

Congruent with the recommendation for the use of CUA in Ghana, QALYs is the preferred health outcome measure. Previous studies in Ghana had used natural units or DALYs as the main outcome measure for health economic evaluations conducted in the country (23;25;29;32). The EQ-5D value set that Ghana has developed is valuable for QALYs adoption as the main health outcome measure in the future. It is noteworthy that, in most high-income countries, QALYs are the single most used health outcome in economic evaluation studies (33). The use of QALYs in Ghana will enhance the cross-comparability of evaluations internally and externally. Given, the fact that there are instances where the use of QALYs may be impossible because of the unavailability of generic instruments to capture QALYs; for example, in situations where interventions for infants and neonates are being evaluated (12), the Ghanaian reference case makes provision for use of DALYs.

One of the key areas of this reference case is the focus on the study perspective and type of costs. This is important to highlight because it remains very context-specific and the need to contextualize the cost and perspective of the analysis cannot be overemphasized. A review of international guidelines in various countries recommends using the healthcare system or payer perspective in analysis, in contrast, the societal perspective is recommended in the case of Ghana. This is so, for two reasons. In Ghana, there is a burgeoning national health insurance scheme (NHIS) that has become a major healthcare financing mechanism in the country. This payer system over the last two decades has paid for healthcare services for all eligible active members who access care in the country. Even though, it is a major healthcare financing scheme in the country, not all of the eligible population are enrolled, as about only 60 percent of the population is enrolled (34). Moreover, there are other services that are currently not covered under the NHIS, so prioritizing the healthcare payer perspective over societal will not be ideal in the context of Ghana. Using the societal perspective will ensure that relevant direct and medical and non-medical costs borne by both the healthcare system and patients are included (12). Further, the recommended societal perspective will ensure that all relevant costs are captured irrespective of who pays for them. In essence, the broader perspective will ensure that the multi-payer system in Ghana is well accounted for, as against the single-payer system in other high-income countries such as the United Kingdom. In fact, in Ghana, depending on the disease or intervention under consideration, financing may be coming from external donors, for example, vaccination, malaria control, etc. so it is appropriate to use the societal perspective so that all relevant costs are accounted for.

Throughout the development process of this reference case, using multi-methods approach, we found that, only a part of the work could be based on international standards and guidelines. For example, for some of the elements such as discount rate, time horizon, transparency, and heterogeneity, the context did not matter so much and adopting international guidelines was just fine. The three percent discount rate is based on common international practice with over 85 percent of previous economic evaluation studies with a time horizon of at least three years covered in the Global Health Cost-effectiveness Registry adopting this rate for both costs and health outcomes (35). We align with this discount rate because of the high volatility nature of the Ghanaian economy which makes it a bit difficult to determine a Ghana-specific discount rate. Notwithstanding, a range of 0–10 percent has been recommended for sensitivity analysis. Also, as already highlighted for health outcomes, study perspectives, and budget impact, it was necessary to contextualize this to suit the needs of Ghana. For example, budget impact is recommended as an integral part of every economic evaluation conducted in Ghana. The reference case recommends that this should be done from the perspective of the NHIS and should be for up to five years. The NHIS has been a healthcare financing method in Ghana since 2003 and remains at the center of the drive for the country to achieve universal health coverage. In fact, it remains in the discussions of almost all health system-related matters of the country including the most recent health financing strategy for the country (2023–2030) (36). It is also imperative to highlight that, the five years was recommended against the backdrop of the political context of Ghana, where national presidential and parliamentary elections are held every four years. This was critically considered because we acknowledge the implications that a change of government has on health financing decisions in the country.

The rigorous multi-method approach used in this study is considered as a key strength for the development of this Ghanaian reference case. We believe the five-throng approach used makes it more rigorous (12). Moreover, this is not a mere recommendatory guideline but provides an academic perspective that details guidance for researchers. Throughout the reference case development process, the research team recognized the peculiarities of the Ghanaian context and allowed some flexibility in special situations.

## Conclusion

The Ghana reference case provides a comprehensive framework for the development, conduct, and reporting of economic evaluations for HTA in Ghana. It details the description of the HTA process, including the steps required for conducting a comprehensive assessment, and the principles of good practice for reporting the outputs.

The reference case offers valuable insights and guidance for policymakers, healthcare practitioners, and researchers in Ghana who are involved in the development and implementation of HTA. By following the recommendations set out in this reference case, Ghana can establish a rigorous and transparent system for evaluating healthcare interventions and technologies to support decision-making which can improve resource allocation, enhance fair pricing, streamline benefit packages for health, and ultimately improve the quality and efficiency of healthcare delivery in the country.

## Data Availability

All information pertaining to the article has been provided in the main text.

## Footnotes

1 (https://yhec.co.uk/glossary/reference-case/)
